# Unique Presentation of a Ubiquitous Organism: Capnocytophaga canimorsus Meningitis With Pneumocephalus

**DOI:** 10.7759/cureus.59529

**Published:** 2024-05-02

**Authors:** Niyati Grewal, Katherine Hager, Anna C Pinelo, Yashmith Duddukunta, Gezahegn G Tolla, Huda Gasmelseed

**Affiliations:** 1 Internal Medicine, Howard University Hospital, Washington DC, USA; 2 Infectious Disease, Howard University Hospital, Washington DC, USA; 3 Internal Medicine, University of South Florida, Tampa, USA; 4 Microbiology, Howard University Hospital, Washington DC, USA

**Keywords:** capnocytophaga meningitis, gram-negative bacteremia, infectious pneumocephalus, adult bacterial meningitis, capnocytophaga canimorsus

## Abstract

*Capnocytophaga canimorsus* is a bacterium commonly found in the oral cavities of cats and dogs. Infections are particularly common in immunocompromised patients who have been exposed to bites or come in contact with saliva from these animals. The manifestations of infection include bacteremia, fever, and, rarely, meningitis. Diagnosis is challenging given the bacteria has slow growth on culture media. The organism is susceptible to beta-lactam antibiotics, with higher-generation cephalosporins recommended for treating meningitis. We present a case of a 74-year-old woman with altered mental status and no signs of immunosuppression. She was diagnosed with meningitis caused by *Capnocytophaga*, with an intriguing finding of pneumocephalus, which is a rare occurrence as per literature review.

## Introduction

*Capnocytophaga canimorsus* is a bacterium commonly found in the oral cavities of cats and dogs. Infections in humans occur when a penetrating wound or abrasion comes in contact with animal saliva [1-4]. These infections are particularly common in immunocompromised patients. Manifestations include bacteremia, fever, and, rarely, meningitis. The diagnosis of this slow-growing organism can be challenging. We present a case of a 74-year-old woman with altered mental status and pneumocephalus, a rare complication of *Capnocytophaga *meningitis.

## Case presentation

**Figure 1:**
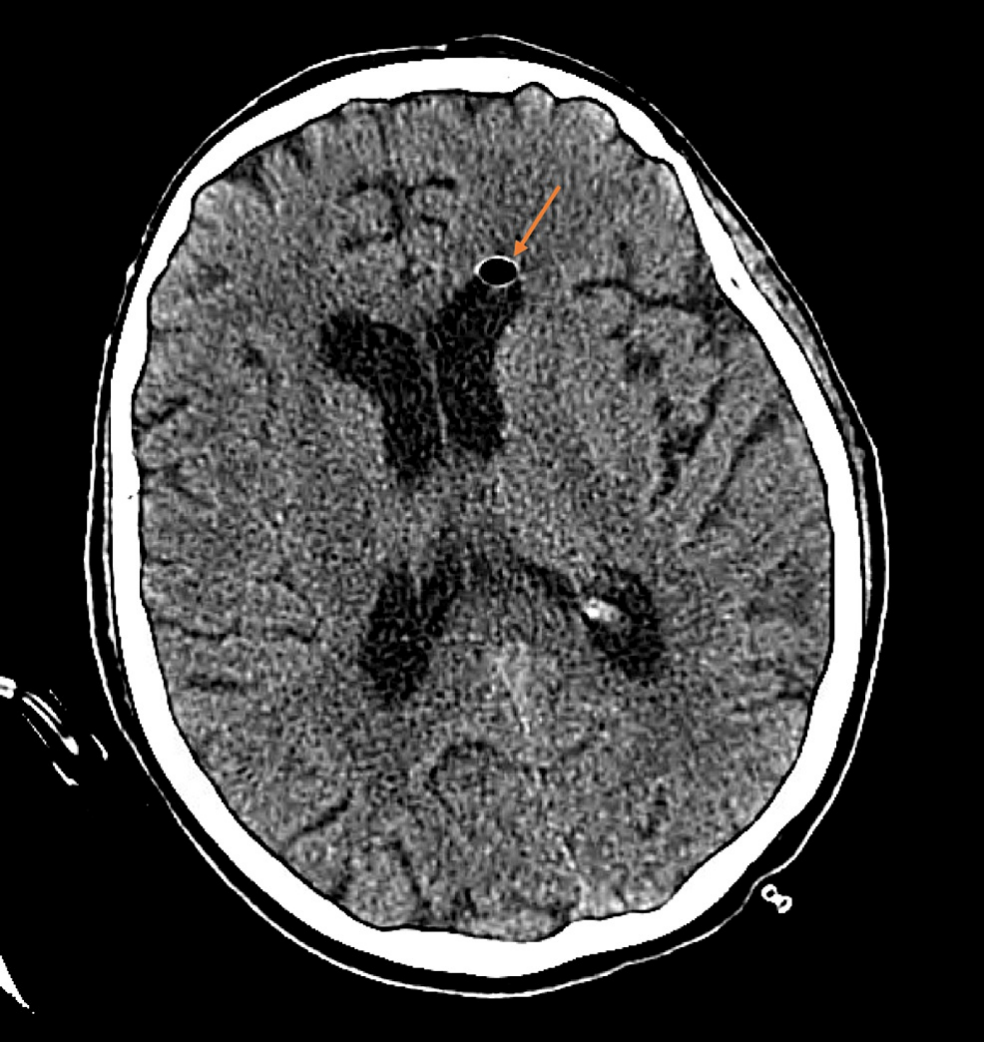
Computed tomography (CT) of the head without contrast shows pneumocephalus in the left lateral ventricle marked by an arrow.

A 74-year-old Caucasian female was brought to the emergency department with altered sensorium after her car was seen swerving off the road. She was found minimally responsive and febrile by the emergency medical services. Initially, the patient was unable to provide a history, but chart review revealed a medical background that included hypertension, hypothyroidism, and scoliosis. On presentation, she had a temperature of 102 degree Fahrenheit, a heart rate of 87 beats per minute, a respiratory rate of 16 breaths per minute, a blood pressure of 148/83 mmHg, and 95% saturation on 3 liters of oxygen via nasal cannula. On physical examination, she appeared disheveled and showed no signs of acute distress. Neurological examination revealed a Glasgow Coma Scale (GCS) of 10 out of 15 (eye opening 3, verbal response 2, motor response 5). Additionally, she exhibited positive Brudzinski and Kernig sign. The extremity exam indicated that her left hand was erythematous and mildly swollen up to the wrist, with a tiny scab but no other rashes. All other system examinations were within normal limits. Initial blood investigations revealed a total leukocyte count of 13.06 x 10^9, with a neutrophil count of 84.9%. Serology for human immune-deficiency virus was non-reactive. The comprehensive metabolic panel was within the normal range. The patient was started on empiric antibiotics and antivirals for suspected meningitis and encephalitis, including vancomycin, ceftriaxone, ampicillin, and acyclovir. A computed tomography (CT) scan of the head without contrast revealed pneumocephalus in the left lateral ventricle (Figure 1).

The antibiotics were changed to vancomycin and meropenem to cover anaerobes and *Listeria.* The cerebrospinal fluid (CSF) analysis revealed an opening pressure of 17 mmHg (normal 10-20 mmHg), glucose <10 mg/dL (normal 40-80 mg/dL), total protein 747 mg/dL (15-60 mg/dL), red blood cells 200/mm^3^ (0-20/mm^3^), total nucleated cells 1487, lymphocytes 7%, and monocytes 2%. Other CSF tests, including *Herpes simplex* virus polymerase chain reaction, venereal disease research laboratory test, West Nile antibody enzyme-linked immunosorbent assay, lyme serology, and cultures, were negative. There was no evidence of immunosuppression based on laboratory tests or patient history. A CT scan of the abdomen from a prior admission revealed a normal spleen.

**Figure 2:**
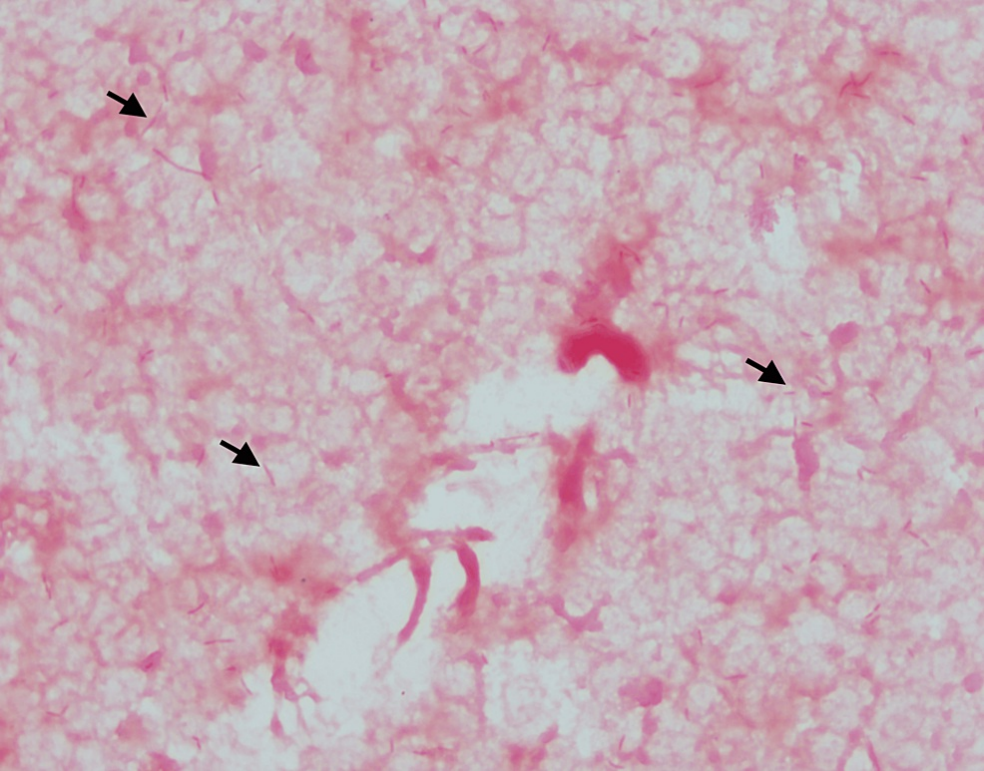
Arrows showing gram-negative fusiform rods with pointed edges on Gram stain from blood culture.

On day 3, the blood culture came back positive, and the gram stain showed gram-negative fusiform rods with pointed edges (Figure 2).

**Figure 3:**
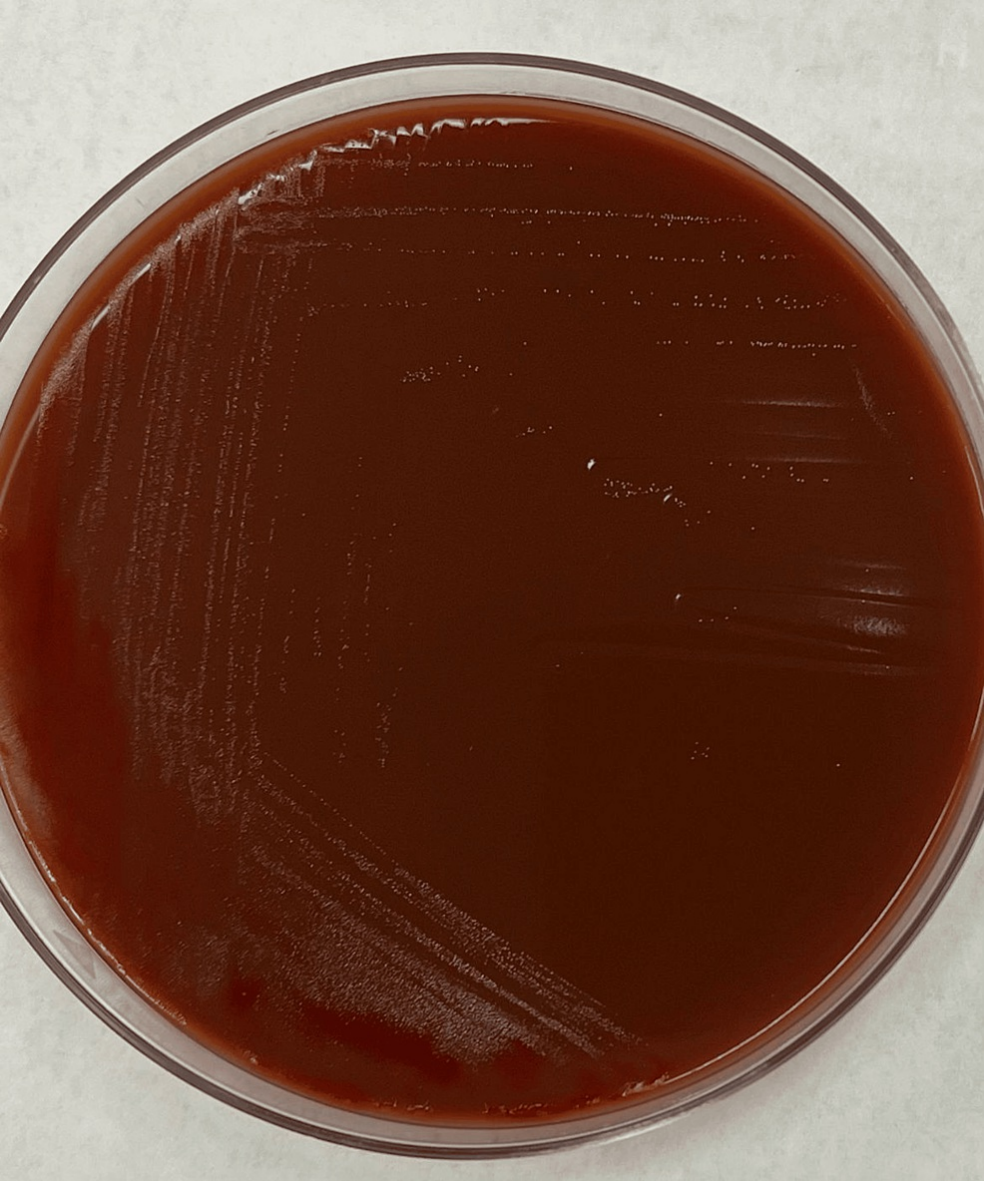
Pinpoint colonies on chocolate agar.

Blood agar showed growth and chocolate agar showed pinpoint colonies (Figure 3).

No growth was seen on MacConkey agar. BioFire Blood Culture Identification (BCID) did not detect the target. Matrix-assisted laser desorption ionization time-of-flight mass spectrometry (MALDI-TOF) identified the organism as *Capnocytophaga canimorsus*. Repeat neuroimaging (CT head) showed resolution of the pneumocephalus, and repeat blood cultures were negative. The patient improved symptomatically, with improvements in mental status, hand wound, and swelling. Further history was obtained from the patient, who mentioned having a pet dog and being bitten by this dog 1 week prior to admission. She was discharged on a 14-day course of amoxicillin-clavulanic acid after nine days of intravenous (IV) antibiotics. 

## Discussion

*Capnocytophaga* is a facultative anaerobe, slow growing, capnophilic, and gram-negative bacilli. It is oxidase and catalase positive anSources of isolates were blood (88%), cerebrospinal fluid (7%), both blood and cerebrospinal fluid (4%), and respiratory tract (2%) [3]. A review by Janda et al. [3] reported that 20% patients survived and 10% died.d ferments glucose, lactose, and maltose [1,2]. It moves slowly with gliding motion, producing spreading edges and finger-like projections at the periphery of the colonies [2]. It is commonly found in the oral cavities of canine and feline. Some species have been isolated from human oral mucosa (Table 1) [1,2].

**Table 1:** Species of Capnocytophaga [1,2]

Species in animal oral mucosa (cats and dogs)	Species in human oral mucosa
C.canimorsus, C. cynodegmi	*C. gingivalis, C. granulosa, C. haemolytica, C. leadbetteri, C. ochracea, C. sputigena, C. genospecies *

*Capnocytophaga* is not a nationally notifiable disease in the United States, and hence no national data is available on the incidence. Literature reviews have reported most cases in men, with a median age of 58 years and a case fatality rate of 33% [3]. Infections in humans occur when a penetrating wound or abrasion comes in contact with animal saliva [1,4]. Some cases have reported eye infections resulting from face licking by animals [4]. The incubation period is about three days or less, with shorter incubation periods due to high infectious doses [1,4]. Risk factors for *Capnocytophaga* infection include immunocompromised status, including but not limited to human immunodeficiency virus, malignancies, immunosuppressive medications, post-organ transplants, alcoholism, asplenism, smoking, corticosteroid therapy, hematological diseases, diabetes, and renal diseases [1,4]. Certain professions exposed to canine and felines, such as veterinarians, and even advanced age, are considered risk factors in some case reports [1,4]. A retrospective review conducted across three centers in the United States between the years 2010 and 2020 highlighted the characteristics of patients with *Capnocytophaga* infection. The median age was 59 years, with 45% of population being males. Comorbidities were identified in the following order of percentage: active immunosuppressive medications (28%), diabetes (15%), hematopoietic stem cell transplantation (13%), active hematologic malignancy (12%), active solid organ malignancy (7%), end-stage renal disease (4%), alcohol use disorder (3%), splenectomy (3%), and solid organ transplantation (3%) [5]. Risk factors for severe disease and poor prognosis include bloodstream infection, sepsis, septic shock, hematological and other malignancies, splenectomy, immunosuppressive therapy, immunosuppression due to other causes, and post-organ transplant [4]. Clinical presentations of *Capnocytophaga canimorsus *infection include sepsis (32%), fever of unknown origin (13%), meningitis (13%), cellulitis (11%), septic shock (9%), respiratory tract infection (7%), phlebitis (2%), endocarditis (2%), urosepsis (2%), septic knee (2%), diverticulitis (2%), and meningococcemia (2%) [3]. Sources of isolates were blood (88%), cerebrospinal fluid (7%), both blood and cerebrospinal fluid (4%), and respiratory tract (2%) [3]. A review by Janda et al. [3] reported that 20% patients survived and 10% died.

*Capnocytophaga* meningitis has an annual incidence reported as 0.03 per 1 million adults in the United States, which is still considered underdiagnosed due to antibiotic prophylaxis that patients receive for dog bites [6]. In literature reviews, only 33 cases have been described with *Capnocytophaga* meningitis, making it a rare manifestation [4]. Specific characteristics of *Capnocytophaga* meningitis were observed to be more common in men, with longer incubation periods and lower mortality [4]. 

Diagnosis can be challenging because this is a slow-growing organism. BioFire and Microscan can miss the organism. The bacteria will grow in the culture media with 5% sheep blood agar or chocolate agar, incubated in 5-10% carbon dioxide at 37 degree Celsius. Classical lab tests are unreliable, and CSF culture on basic media gives many false-negative results [1,4-7]. Molecular tests like CSF polymerase chain reaction (PCR) and 16 small ribonucleic acid (sRNA) gene sequencing are also useful. MALDI-TOF mass spectrometry was the test that diagnosed the infection in our patient and is often a reliable modality. *Capnocytophaga canimorsus* is susceptible to beta-lactams. Higher-generation cephalosporins like ceftriaxone or cefepime are advised for meningitis due to their ability to produce beta-lactamases [1-2,4-6]. From literature reviews, mild infections were treated with clindamycin, doxycycline, amoxicillin/clavulanic acid, and fluoroquinolones. Carbapenems are used for mixed soft tissue infections or multi-resistant strains [1-2,4-6]. Aminoglycosides, anti-staphylococcal penicillins, colistin, and trimethoprim-sulfamethoxazole were found to be ineffective or shown to have resistance. The duration of treatment is unclear, with most reports mentioning treatment given for 7-10 days, showing improvement in clinical status. Some case reports recommend giving amoxicillin/clavulanic acid for prophylaxis after a dog bite. 

Pneumocephalus is defined as the presence of air in the epidural, subdural, or subarachnoid space within the brain parenchyma or ventricular cavities [8]. It can occur spontaneously or after trauma, cranial surgeries, or infections. Infections associated with pneumocephalus are usually meningitis or ventriculitis with gas-producing organisms [8]. Diagnosis is made by CT head or magnetic resonance imaging of the brain. Treatment is usually conservative, involving bed rest, placing the patient in 30-degrees Fowler position, avoiding Valsalva maneuvers, and providing high-flow oxygen. Literature suggests that air composed of 78% nitrogen and 21% oxygen enhances the resorption of pneumocephalus [8]. Surgical treatment is used in patients who are symptomatic, have increased intracranial pressure, recurrent pneumocephalus, or if it persists for more than one week. Surgical options include needle aspiration, ventriculostomy, and decompressive craniectomy [8].

Pneumocephalus in meningitis with* Capnocytophaga* is rare, and our literature review didn’t show any reported cases. Organisms associated with meningitis and pneumocephalus include *Clostridium septicum, perfringens, Acinetobacter sp, Streptococcus pneumoniae, Streptococcus pyogenes*, and *Bacteroides fragilis* [9-12]. Cases with pneumocephalus and meningitis caused by other organisms showed spontaneous resolution within 48 hours, which was also observed in our case.

## Conclusions

*Capnocytophaga* infections are most commonly seen in immunocompromised patients. Our patient didn’t have any obvious evidence of immunosuppression, making the presentation unusual. Pneumocephalus is also an unusual presentation with *Capnocytophaga *infection. Infections are usually underdiagnosed due to slow bacterial growth on culture media. Meningitis is a rare presentation of *Capnocytophaga canimorsus*, but most cases from literature review responded well to treatment, and mortality was low. Our aim is to highlight the unusual presentation and response to treatment in our patient with *Capnocytophaga canimorsus,* with a scope for future studies on the etio-pathogenesis, prevention, and treatment of infections with this organism.
